# Costs for Long-Term Health Care After a Police Shooting in Ontario, Canada

**DOI:** 10.1001/jamanetworkopen.2023.35831

**Published:** 2023-09-28

**Authors:** Sheharyar Raza, Deva Thiruchelvam, Donald A. Redelmeier

**Affiliations:** 1 Department of Medicine, University of Toronto, Toronto, Ontario, Canada; 2 Evaluative Clinical Sciences, Sunnybrook Research Institute, Toronto, Ontario, Canada; 3 Institute of Clinical Evaluative Sciences (ICES) in Ontario, Ontario, Canada; 4 Institute for Health Policy Management and Evaluation, Ontario, Canada; 5 Division of General Internal Medicine, University of Toronto, Toronto, Ontario, Canada

## Abstract

**Importance:**

Police shootings can cause serious acute injury, and knowledge of subsequent health outcomes may inform interventions to improve care.

**Objective:**

To analyze long-term health care costs among survivors of police shootings compared with those surviving nonfirearm police enforcement injuries using a retrospective design.

**Design, Setting, and Participants:**

This population-based cohort analysis identified adults (age ≥16 years) who were injured by police and required emergency medical care between April 1, 2002, and March 31, 2022, in Ontario, Canada.

**Exposure:**

Police shootings compared with other mechanisms of injury involving police.

**Main Outcomes and Measures:**

Long-term health care costs determined using a validated costing algorithm. Secondary outcomes included short-term mortality, acute care treatments, and rates of subsequent disability.

**Results:**

Over the study, 13 545 adults were injured from police enforcement (mean [SD] age, 35 [12] years; 11 637 males [86%]). A total of 13 520 individuals survived acute injury, and 8755 had long-term financial data available (88 surviving firearm injury, 8667 surviving nonfirearm injury). Patients surviving firearm injury had 3 times greater health care costs per year (CAD$16 223 vs CAD$5412; mean increase, CAD$9967; 95% CI, 6697-13 237; US $11 982 vs US $3997; mean increase, US $7361; 95% CI, 4946-9776; *P* < .001). Greater costs after a firearm injury were not explained by baseline costs and primarily reflected increased psychiatric care. Other characteristics associated with increased long-term health care costs included prior mental illness and a substance use diagnosis.

**Conclusions and Relevance:**

In this longitudinal cohort study of long-term health care costs, patients surviving a police shooting had substantial health care costs compared with those injured from other forms of police enforcement. Costs primarily reflected psychiatric care and suggest the need to prioritize early recognition and prevention.

## Key Points

**Question:**

When compared with other mechanisms of injury involving police, are police shootings associated with greater long-term health care costs?

**Findings:**

In this population-based cohort study with data for 13 545 adults injured from police intervention, there was a 3-fold increase in long-term annual health care costs for patients surviving a police shooting compared with other mechanisms of injury. Most of the increased costs were due to psychiatric care.

**Meaning:**

This study found a substantial increase in the health care costs in the years after a police shooting, thereby highlighting the need for early multimodal interventions.

## Teaser

This cohort study examines follow-up data for adults injured by police to analyze long-term health care costs among survivors of police shootings compared with those surviving nonfirearm police enforcement injuries.

## Introduction

Police shootings are defined as injuries involving a firearm discharge by law enforcement agents while on duty.^1^ Police shootings often reflect split-second decisions, lead to about a thousand deaths in the United States annually, and garner extensive public media attention through local and national reporting.^2,3^ However, most police enforcement injuries do not involve a firearm and do not cause death.^4,5^ Ultimately, many civilians survive injury with uncertain degrees of disability and potentially accrue long-term health care costs.^6,7,8,9^

Public discourse around police shootings sometimes neglects the extended aftermath. First, most police shootings are not fatal, and death counts alone underestimate the extent of personal losses.^10^ Second, modern acute care is increasingly effective, so declining mortality trends might not mirror the collective burden of losses.^11^ Third, the extent of injury is not always initially apparent.^12^ Fourth, other mechanisms of police enforcement are not always innocuous.^13,14^ Fifth, the extent of long-term disability for survivors is often uncertain at hospital discharge.^15,16,17^ Sixth, the larger goal of restoring productive members to society requires many personal, medical, and social resources.^18^

The mean cost of acute care for a patient with a gun injury in Canada is about CAD$32 203 (US $17 593) from emergency transport, initial resuscitation, and critical care.^8,19^ However, long-term costs are substantially larger and include lost employment, personal damages, and other intangible effects.^20^ For police shootings, little is known about the long-term economic and health outcomes for survivors. The purpose of this study was to evaluate the long-term health care costs and disability for adults surviving a police shooting.

## Methods

### Setting

Ontario is Canada’s most populous province and accounts for the most police shootings in the country.^21,22^ The mean health care cost is CAD$4883 (US $3609) per person annually. The annual population incidence of shootings in Ontario is about 400 to 500, of which approximately 2% to 3% involve the police (including police shot in the line of duty).^6,23,24,25^

Prevailing laws during the study mandated medical reporting of all patients with a gun injury.^26^ Emergency care was universally available, publicly funded by the Ontario Health Insurance Plan (OHIP), and tracked through encrypted linked records after a lag for data security and collection.^27,28^ Patient-level linked health services data were available to qualified researchers through population-based electronic medical records at the Institute for Clinical Evaluative Sciences (ICES).^29,30,31,32,33^

The study protocol covering privacy, security, and ethics was approved by the Sunnybrook Research Ethics Board, including a waiver for direct patient consent. The study was also approved by ICES with standard privacy and security safeguards. The use of the data in this project was authorized under section 45 of Ontario’s Personal Health Information Protection Act. The study design and reporting followed the Strengthening the Reporting of Observational Studies in Epidemiology (STROBE) reporting guideline and ICES standards.^34^

### Police Shootings

We identified consecutive adults (age ≥16 years) injured in a police shooting who received emergency care between April 1, 2002, and March 31, 2022. Past reports established that available databases were comprehensive (covering >99% of emergency departments), connected (linkage rates >95%), and consistent (diagnostic reliability >90% compared with medical record abstraction).^29,35,36^ Patients were identified using codes from the *International Statistical Classification of Diseases, Tenth Revision, Clinical Modification (ICD-10-CM),* as validated in past research (codes Y35.0 to Y35.99).^37,38,39,40,41,42^ We excluded patients who died at the scene, those living outside Ontario, individuals lacking a valid health card number, and youth younger than 16 years. Patients with more than 1 incident were analyzed by the first presentation to avoid statistical artifacts from double counting due to trauma recidivism (eFigure in Supplement 1).^43^

Diagnostic codes identified injuries from police enforcement as diagnosed by clinicians. We defined a police shooting as an injury “caused by legal intervention involving a firearm discharge” (code Y35.0). This included revolvers, rifles, rubber bullet weapons, machine guns, or other firearm types. We defined control injuries as those caused by other forms of police enforcement that also required emergency care. This included incidents involving batons, staves, blunt objects, manhandling, tear gas, electricity, asphyxiation, explosives, bayonets, or unspecified means (codes Y35.1 to Y35.99). A limitation of this approach was the lack of data on injuries indirectly related to police enforcement such as a traffic crash after a police chase. A further limitation is that each of the 2 groups had diverse types of injuries because of the range of potential mechanisms.

### Additional Characteristics

Data on patient age (years), socioeconomic status (quintile), sex (binary), and home (urban, rural) were based on linked demographic databases.^44,45^ Additional health care records identified the incident time (day and hour).^46^ Uncertain cases were classified as nonfirearm injuries so that no patient was excluded from analysis. Additionally, we identified psychiatric illnesses and substance use disorders in the year before the injury from linked outpatient medical records.^47,48^ We obtained additional indicators of overall utilization in the year before injury to calculate baseline health care costs (hospital, emergency, outpatient).^49^ Information on crime records, educational attainment, ethnic background, adverse childhood experiences, and military service was not available.

### Acute Care

We examined short-term acute care clinical outcomes for context and comparison with past research from other regions.^12,50,51^ Prehospital variables included time of arrival, day of injury, and use of ambulance transport. Emergency care indicators included surgical procedures, blood transfusions, and critical care (each as a separate binary indicator). Hospital mortality included patients who died in the emergency department, during initial resuscitation, later during the hospital stay, or after transfer to a specialized acute trauma center. Hospital length of stay was determined as total time in days from arrival in an emergency department until death or hospital discharge.

### Subsequent Disability

Chronic disability was defined by the formal submission of a disability support application as determined using official social service records (OHIP codes K050-K054).^52^ Long-term disability support applications in this setting required a medical report by a responsible physician (Health Status Report, Activities of Daily Living Index, Special Necessities Benefit Form).^53^ An application reflected the patient’s perception of disability and required physician authentication. This methodology has been validated in past research and may underestimate the burden of disability.^54^ We considered death as a competing outcome for analysis of long-term disability. The available databases did not contain information on the specific nature of the disability, whether a formal application was rejected, or how a disability connected to the original injury.

### Primary Outcome

We applied the ICES long-term costing algorithm to estimate the total direct annual costs of health care following the initial injury adjusting for inflation (reference year 2021).^55,56^ This methodology has been validated in past research of trauma patients.^57,58,59,60,61^ For the cost analysis, we restricted the analysis to complete cases with at least 5 years of available follow-up data (even if some intervals involved zero costs). Each analysis was concluded at 5 years to minimize biases from uneven durations of longer patient follow-up. These restrictions allowed economic estimates to provide unbiased analyses of annual costs over a 5-year longitudinal follow-up (yet underestimated the total lifetime costs of injury). Two further assumptions were that medication costs were not included (not consistently available) and ancillary costs from insurance, unemployment, bankruptcy, or incarceration could not be evaluated (also unavailable). Costs were expressed in prevailing Canadian dollars and may differ from US charges.^62^

### Specific Cost Components

Health care costs were estimated by combining institutional expenses, physician payments, and miscellaneous services. Institutional expenses included mental health services, medical care, surgical procedures, rehabilitation treatment, chronic care, outpatient clinic, and same-day surgery costs. Physician payments were based on the prevailing payment schedule and included general fee-for-service, specialist fee-for-service, and physicians practicing in prisons.^56,63^ Miscellaneous services included home care service, emergency visits, and outpatient miscellaneous costs. Nonphysician emergency department costs were estimated according to Comprehensive Ambulatory Classification Systems groups.^64^ These estimation methods have been validated in past research.^65,66,67,68^

### Statistical Analysis

The primary analysis tested whether annual health care costs were higher for patients after police shootings compared with control patients injured by other mechanisms involving police enforcement. The follow-up interval began on the day of hospital discharge, excluded patients who did not survive initial injury, and examined a time horizon of 5 years (expressed as annual costs). We also used a self-matched design to evaluate annual costs for each patient before and after injury (identifying each person as their own control and quantifying relative cost ratios with corresponding geometric means). Secondary analyses evaluated rates of acute mortality (unadjusted cumulative incidence computed using logistic regression to analyze death rates) and rates of disability (using the Fine and Gray model for subdistributional hazard ratios). No data imputation methods were used.

Additional analyses were conducted for descriptive and exploratory purposes. We profiled baseline demographic and medical characteristics for the 2 groups to assess differences between patients injured by police shootings vs those with control injuries (eTable 1 in Supplement 1). We examined basic elements of acute treatment to profile initial medical care. We used multivariable regression models to explore additional independent correlates of long-term health care costs. We unbundled subsequent expenditures by category to identify the major contributors to long-term health care costs.

## Results

A total of 13 545 adults were injured by police enforcement during the 20-year study, a mean of 2 individuals per day. The typical patient in each group was a man younger than 40 years who was living in a city at or below the middle socioeconomic level (Table 1). Few patients were injured by police shootings (n = 178) and most patients by other mechanisms (n = 13 367). Those injured by police shootings were relatively more likely to be male, living in a rural region, and previously diagnosed with a mental health condition. Mean (SD) health care costs for a patient during the year before injury showed no significant difference between the 2 groups (CAD$5384 [10 094] vs CAD$5179 [15 457]; US $3976 [7455] vs $3825 [11 416]; *P* = .77).

**Table 1.** Baseline Patient Characteristics

Characteristic	No. (%)	
	Firearm (n = 178)^a^	Control (n = 13 367)^b^
Age^c^		
≤29 y	69 (39)	5435 (41)
≥30 y	109 (61)	7932 (59)
Sex		
Male	166 (93)	11 471 (86)
Female	12 (7)	1896 (14)
Home		
Urban	150 (84)	11 952 (89)
Rural	28 (16)	1415 (11)
Socioeconomic quintile^d^		
Highest	19 (11)	1514 (11)
Next to highest	24 (13)	1921 (14)
Middle	24 (13)	2326 (17)
Next to lowest	52 (29)	2819 (21)
Lowest	59 (33)	4787 (36)
Timing of acute incident		
Nighttime		
Yes	69 (39)	5061 (38)
No	109 (61)	8306 (62)
Weekend		
Yes	50 (28)	4501 (34)
No	128 (72)	8866 (66)
Past-year health care		
Mental health diagnosis^e^		
Yes	86 (48)	5359 (40)
No	92 (52)	8008 (60)
Substance misuse diagnosis^f^		
Yes	33 (19)	2633 (20)
No	145 (81)	10 734 (80)
≥7 Outpatient visits		
Yes	48 (27)	4281 (32)
No	130 (73)	9086 (68)
Emergency visit		
Yes	96 (54)	7267 (54)
No	82 (46)	6100 (46)
Hospital admission		
Yes	21 (12)	1248 (9)
No	157 (88)	12 119 (91)
Total health care costs, CAD$^g^		
Mean (SD)	5384 (10 094)	5179 (15 457)
Median (IQR)	1105 (271-5672)	953 (248-3870)

^a^ Mechanism of injury was rifle, revolver, machine gun, or other firearm discharge.

^b^ Mechanism of injury was manhandling, blunt object, sharp object, gas, explosive, or unspecified means.

^c^ Median (IQR) age, 33 (25-43) years; mean (SD) age, 34.48 (12.19) years.

^d^ Based on home neighborhood.

^e^ Ontario Health Insurance Plan diagnostic codes 290 to 316 (except 293, 294, 303, 304, 308, 310, 312).

^f^ Ontario Health Insurance Plan diagnostic codes 303 to 304.

^g^ Canadian dollars adjusted to 2021.

### Acute Care

The profile of acute care emergency treatment suggested greater injury severity for patients after a police shooting relative to controls. This discrepancy included a higher frequency of ambulance involvement, hospital admission, surgical procedures, critical care admissions, and blood product transfusions (Table 2). The mean (SD) hospital length of stay was 1 week longer for patients after a police shooting relative to controls (13.5 [18] days vs 5.8 [9] days, respectively; *P* < .001). A total of 25 patients died during initial hospitalization, with higher mortality for patients after a police shooting relative to controls (16 [9.0%] vs 9 [0.1%], respectively; *P* < .001). The remaining 162 patients with firearm injuries and 13 358 patients with control injuries survived the initial hospitalization.

**Table 2.** Acute Care Medical Treatments

Treatment	No. (%)		*P* value
	Firearm (n = 178)	Control (n = 13 367)	
Ambulance			
Yes	122 (69)	3817 (29)	<.001
No	56 (32)	9550 (71)	
Hospital admission			
Yes	51 (29)	320 (2)	<.001
No	127 (71)	13 047 (98)	
Surgical procedure			
Yes	28 (16)	95 (1)	<.001
No	150 (84)	13 272 (99)	
Critical care			
Yes	22 (12)	53 (0.4)	<.001
No	156 (88)	13 314 (99.6)	
Blood transfusion^a^			
Yes	11 (6)	9 (0.1)	<.001
No	167 (94)	13 358 (99.9)	
Time in hospital, d			
Mean (SD)	13.47 (18)	5.76 (9)	<.001
Median (IQR)	7 (3-15)	3 (2-6)	
Outcome^b^			
Death	16 (9)	9 (0.1)	<.001
Alive	162 (91)	13 358 (99.9)	

^a^ Based on packed red cells, platelets, or whole blood.

^b^ Includes emergency or hospital and excludes deaths at the scene.

### Chronic Disability

The 13 520 total survivors accounted for 52 102 patient-years of follow-up (mean, 3.85 years). Patients surviving a police shooting accounted for 28 subsequent cases of disability over 525 patient-years of follow-up, equal to an incidence of 53 per 1000 patients annually. Patients surviving control injuries accounted for 2181 subsequent cases of disability over 51 577 patient-years of follow-up, equal to an incidence of 42 per 1000 patients annually. Police shootings were associated with a 21% relative increased incidence of subsequent disability that was not statistically significant (95% CI, −17% to 76%). For both groups, substantial numbers of cases of disability became apparent after the first year (Figure).

**Figure.**
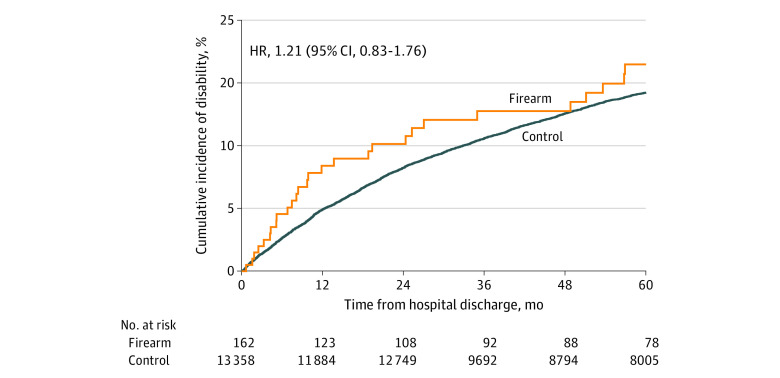
Risk of Long-Term Disability Cumulative incidence plots of absolute risk of disability after injury. The upper line indicates patients injured from a police shooting. The control curve indicates patients injured by other mechanisms. Results show increasing incidence of disability with time and no significant difference after a police shooting compared with other mechanisms of injury. Estimated hazard ratio (HR) is based on the Fine and Gray model.

### Subsequent Health Care Costs

Long-term costs totaled CAD$241 675 273 (US $178 491 690) among the 8755 long-term survivors who had 5 years of follow-up data available (eTable 2 in Supplement 1). Patients surviving a police shooting had mean costs of CAD$16 223 (US $11 982) annually whereas patients with control injuries had costs of CAD$5412 (US $3997) annually. The net difference unadjusted for baseline costs equaled an increase of CAD$10 811 annually (95% CI, 1605-20 017; US $7985; 95% CI, $1185-$14 784; *P* = .02). Adjusted analysis matching annual costs before and after injury for each individual indicated a 3.96 relative increase for survivors of police shootings (95% CI, 2.71-5.78) and a 2.06 relative increase for controls (95% CI, 2.00-2.13). The net differential was equal to a further relative increase of 1.90 after firearm injuries compared with control injuries (*P* < .001).

Secondary analyses of survivors examined specific components of care contributing to the differences in long-term health care costs. The largest single contributor was inpatient psychiatric care costing a mean (SD) of CAD$10 573 (42 981) annually (mean [SD], US $7809 [31 744]) after a police shooting and CAD$1348 (12 275) annually (mean [SD], US $996 [9066]) after a control injury (Table 3). The next largest contributors were outpatient clinics and home care services. The difference in total costs was not explained by surgical inpatient care, emergency visits, or outpatient physician services. If patients who survived a police shooting had costs that instead followed the patterns of those for control patients, we estimated a potential savings of CAD $1.88 million (US $1.39 million) in health care costs.

**Table 3.** Components of Long-Term Annual Costs^a^

Component	Firearm (n = 88)^b^	Control (n = 8667)	*P* value^c^
Total			
Mean (SD)	16 223 (43 422)	5412 (15 588)	<.001
Median (IQR)	3468 (1206-10 589)	1480 (565-4534)	<.001
Institutional			
Inpatient mental health			
Mean (SD)	10 573 (42 981)	1348 (12 275)	<.001
Median (IQR)	0 (0-174)	0 (0-0)	<.001
Inpatient medical or surgical			
Mean (SD)	1321 (3446)	873.84 (3521)	.24
Median (IQR)	189 (0-968)	0 (0-222)	<.001
Inpatient rehabilitation or chronic care^d^			
Mean (SD)	213 (1021)	152 (2800)	.84
Median (IQR)	0 (0-0)	0 (0-0)	.008
Hospital outpatient clinic			
Mean (SD)	809 (1399)	418 (934)	<.001
Median (IQR)	267 (38-790)	91 (0-406)	<.001
Same-day surgery			
Mean (SD)	121 (301)	85 (245)	.17
Median (IQR)	0 (0-46)	0 (0-0)	.24
Physician			
Generalist fee-for-service			
Mean (SD)	416 (680)	387 (1132)	.810
Median (IQR)	217 (78-478)	154 (59-366)	.08
Specialist fee-for-service			
Mean (SD)	811 (859)	645 (1147)	.18
Median (IQR)	524 (162-1180)	262 (74-720)	<.001
Other physician plans^e^			
Mean (SD)	208 (350)	168 (319)	.24
Median (IQR)	127 (34-252)	95 (32-199)	.14
Miscellaneous			
Home care service			
Mean (SD)	281 (1458)	82 (702)	.009
Median (IQR)	0 (0-0)	0 (0-0)	.005
Emergency visits			
Mean (SD)	562 (1278)	474.89 (1074)	.45
Median (IQR)	198 (71-506)	213 (95-473)	.64
Outpatient miscellaneous^f^			
Mean (SD)	905.97 (1787)	779.15 (3033)	.70
Median (IQR)	107 (27-754)	71 (16-359)	.05

^a^ Costs are given in Canadian dollars adjusted to 2021.

^b^ Injured in police shootings (n = 178), excluding deaths (n = 16) and those with insufficient financial data (n = 74).

^c^ *P* values for difference in means based on the *t* test and difference in medians based on the Wilcoxon rank.

^d^ Includes National Rehabilitation Reporting System, Continuing Care Reporting System, Ontario Health Insurance Plan, Ontario Drug Benefit, and complex long-term care.

^e^ Includes capitated physicians and non–fee-for-service arrangements.

^f^ Includes dialysis, cancer, allied professionals, and special drug costs.

### Associations With Long-Term Health Care Costs

Supplementary analyses of long-term survivors examined additional characteristics associated with increased health care costs after surviving acute injury. Older age, weekday timing for the incident, and prior health care costs were each associated with higher long-term health care costs (Table 4). A past diagnosis of mental illness or substance misuse was also associated with higher long-term costs (CAD$3694 and CAD$2721, respectively; US $2728 and $2010, respectively). Conversely, patient socioeconomic status and sex showed no significant association. Adjustment for all baseline patient characteristics suggested that a police shooting was associated with significantly higher long-term annual costs relative to controls (mean increase, CAD$9967; 95% CI, 6697-13 237; US $7361; 95% CI, 4946-9776; *P* < .001).

**Table 4.** Predictors of Long-Term Annual Costs^a^

Predictor	Basic analysis, absolute amount, CAD$ (95% CI)^b^	Adjusted analysis, absolute increase, CAD$ (95% CI)^c^
Mechanism of acute injury		
Firearm	16 223 (7022 to 25 423)	9967 (6697 to 13 237)
Control	5412 (5084 to 5740)	1 [Reference]
Age group		
Younger (≤29 y)	4031 (3562 to 4500)	−1272 (−1940 to −604)
Older (≥30 y)	6675 (6200 to 7150)	1 [Reference]
Sex		
Male	5220 (4864 to 5577)	−788 (−1772 to 197)
Female	7534 (6508 to 8561)	1 [Reference]
Home location		
Urban	5644 (5277 to 6011)	1106 (23 to 2190)
Rural	4429 (3711 to 5147)	1 [Reference]
Socioeconomic group		
Higher^d^	5202 (4422 to 5983)	665 (−344 to 1674)
Middle	4823 (4113 to 5533)	1 [Reference]
Lower^e^	5885 (5463 to 6308)	588 (−309 to 1485)
Incident time		
Night	6061 (5452 to 6669)	370 (−307 to 1046)
Daytime	5200 (4800 to 5599)	1 [Reference]
Incident day		
Weekend	4527 (4070 to 4984)	−918 (−1605 to −231)
Weekday	6057 (5599 to 6515)	1 [Reference]
History		
Mental health diagnosis		
Yes	9827 (9054 to 10 599)	3694 (2922 to 4467)
No	3121 (2836 to 3406)	1 [Reference]
Substance misuse diagnosis		
Yes	11 114 (10 074 to 12 153)	2721 (1737 to 3704)
No	4501 (4154 to 4848)	1 [Reference]
Prior health care costs		
≥CAD$1000	9573 (8897 to 10 250)	4493 (3716 to 5270)
<CAD$1000	2371 (2112 to 2629)	1 [Reference]

^a^ Analyses based on all patients surviving acute injury with long-term financial data. Estimates are annual health care costs in Canadian currency.

^b^ No adjustment for baseline differences.

^c^ Adjusted for all measures in univariable analysis.

^d^ Denotes highest or next highest socioeconomic quintile.

^e^ Denotes lowest or next lowest socioeconomic quintile.

## Discussion

We studied data for more than a hundred patients injured from a police shooting and thousands injured from other mechanisms by police enforcement. We found a 3-fold increase in long-term health care costs among those surviving a police shooting relative to controls. The absolute increase in long-term health care costs were substantial, a mean of nearly CAD$10 000 per patient annually for those who survived a police shooting. The increased long-term costs were not explained by demographic characteristics and mostly related to psychiatric care services (with modest differences in other medical care). Together, the findings suggest that police shootings have associations with acute mortality, emergency resuscitation, and long-term health care costs.

### Past Research

Our results support findings from other settings examining police shootings. Studies from the United States suggest that 99% of police-related injuries are not from firearms.^69^ We also found an overrepresentation of young men of lower socioeconomic status living in cities, in accord with other research.^70^ Additionally, we demonstrated high rates of acute mortality, critical care, blood transfusions, and surgical procedures after police shootings comparable with reports from diverse contexts.^71,72^ Our finding of a significant increase in psychiatric care after a shooting corroborates past research on individuals experiencing nonfatal shooting assault.^73^ In line with qualitative research, we found substantial delayed consequences, including ongoing health care costs and long-term disability.^74,75^ Consistent with social science descriptions, we found extensive psychiatric burden for patients who survive a police shooting.^76^

Several reasons might explain why police shootings are associated with substantial long-term health care costs. Psychological theories of trauma-induced disability emphasize a progression after physical injury with initial helplessness followed by chronic maladjustment.^77^ The cascade of physical and psychological losses is supported by studies of patients who develop mental illness in the aftermath of a civilian firearm injury (eg, anxiety, mood disorders, attention deficits).^78,79,80^ Compared with other forms of enforcement, police shootings may exacerbate depression (two-thirds of patients) and posttraumatic stress (half of patients).^81,82^

Psychiatry research involving military combatants suggests early therapy may prevent the emergence of mental illness after gunshot injuries. Randomized clinical trials show that antidepressants may mitigate the emergence of posttraumatic stress disorder in some patients (eg, sertraline, 50 mg once daily).^83^ α-Blockers might potentially lessen nightmares in patients after traumatic exposure (eg, prazosin, 10 mg at bedtime).^84^ Daily light therapy may possibly abate symptoms of seasonal depression (eg, 10 000 lumens for 1 hour).^76,85,86,87^ Together, evidence from military medicine suggests early intervention could potentially mitigate mental illness in survivors of police shootings.^88^

### Future Directions

Additional research could further explore how police shootings are associated with long-term health care costs. An incipient mental health crisis could have provoked reactive police enforcement in the first place, although we found no corresponding imbalance in mental health diagnosis prior to injury.^89,90^ Mistrust of medical institutions might also be common after police shootings and may lead to delays in preventive care or unchecked accumulating morbidity in survivors.^74,75,91^ Death anxiety may develop, whereby survivors become constantly fearful of further disability and exhibit excessive care-seeking.^79^

A police shooting may cause ripple effects throughout communities that weaken social support networks.^92,93,94^ Specific psychiatric interventions also need to be directly tested in this setting to assess effectiveness and safety. In addition, more complicated econometric modeling with data imputation might be considered for testing repeated events or cases with partial follow-up intervals. Chronic pain may lead to prolonged reliance on analgesics or substance use disorders.^95^ These adverse sequelae and risks of recurrence are opportunities for future research.^93,96^

### Limitations

Our study has several limitations. The analysis did not include data on financial expenses not covered by the universal health insurance plan (eg, psychology therapy, private rehabilitation, legal fees, incarceration costs, or lost earnings), patients with minor injuries not requiring medical attention, and those with insufficient follow-up (incomplete cases); therefore, the data may underestimate overall losses.^92^ Economic analyses are further limited by lags in the billing system and other accounting delays that can sometimes require years to resolve and finalize. Follow-up is confined to a 5-year period and underestimates health care costs accrued over longer time horizons, including costs for patients who died prematurely. The analysis did not account for major changes in practice patterns over time or the fallibility of *ICD-10-CM* codes.

The health records did not connect simultaneous shootings that sometimes involve multiple individuals. The costing algorithm did not include health care costs such as personal services (eg, private physiotherapy), material costs (eg, property damage), second survivor costs (eg, witnesses experiencing psychological trauma), or other losses in societal goods and services. Our study reports nothing about police training, staffing, supervision, funding, ammunition, or firearm model.^13,97,98,99^ Our findings did not include data on ethnicity, immigration, criminal record, or whether the use of force was justified.^74,100,101^ The study cannot distinguish whether a mental health crisis incited a police shooting or whether a police shooting exacerbated a mental health crisis. Our design was observational, cannot establish causality, and was based in Canada.

## Conclusions

This study examined long-term outcomes among patients injured by police and found substantial ongoing health care costs. The costs were especially high among those surviving a police shooting. The relatively higher health care cost for adults after a police shooting were not explained by baseline differences in health care and were primarily explained by mental health services. Future research might examine whether treatments studied in military settings are effective for civilians surviving a police shooting.

## Notes

Supplement 1. **eFigure.** Description of Patient Selection **eTable 1.** Description of Full Cohort **eTable 2.** Description of Long-Term Cases

Supplement 2.
Data Sharing Statement
