# Supplementary material for: Costs for Long-Term Health Care After a Police Shooting in Ontario, Canada
Source: JAMA Netw Open. 2023 Sep 28;6(9):e2335831. doi: 10.1001/jamanetworkopen.2023.35831 (PMC10539992; doi:10.1001/jamanetworkopen.2023.35831)
Supplement: Supplement 1. — eFigure. Description of Patient Selection eTable 1. Description of Full Cohort eTable 2. Description of Long-Term Cases [file jamanetwopen-e2335831-s001.pdf]

Supplemental Online Content

Raza S, Thiruchelvam D, Redelmeier DA. Costs for long-term health care after a police shooting in Ontario, Canada. *JAMA Netw Open*. 2023;6(9):e2335831. doi:10.1001/jamanetworkopen.2023.35831

eFigure. Description of Patient Selection
eTable 1. Description of Full Cohort
eTable 2. Description of Long-Term Cases

This supplemental material has been provided by the authors to give readers additional information about their work.

© 2023 Raza S et al. *JAMA Network Open.*

eFigure. Description of Patient Selection

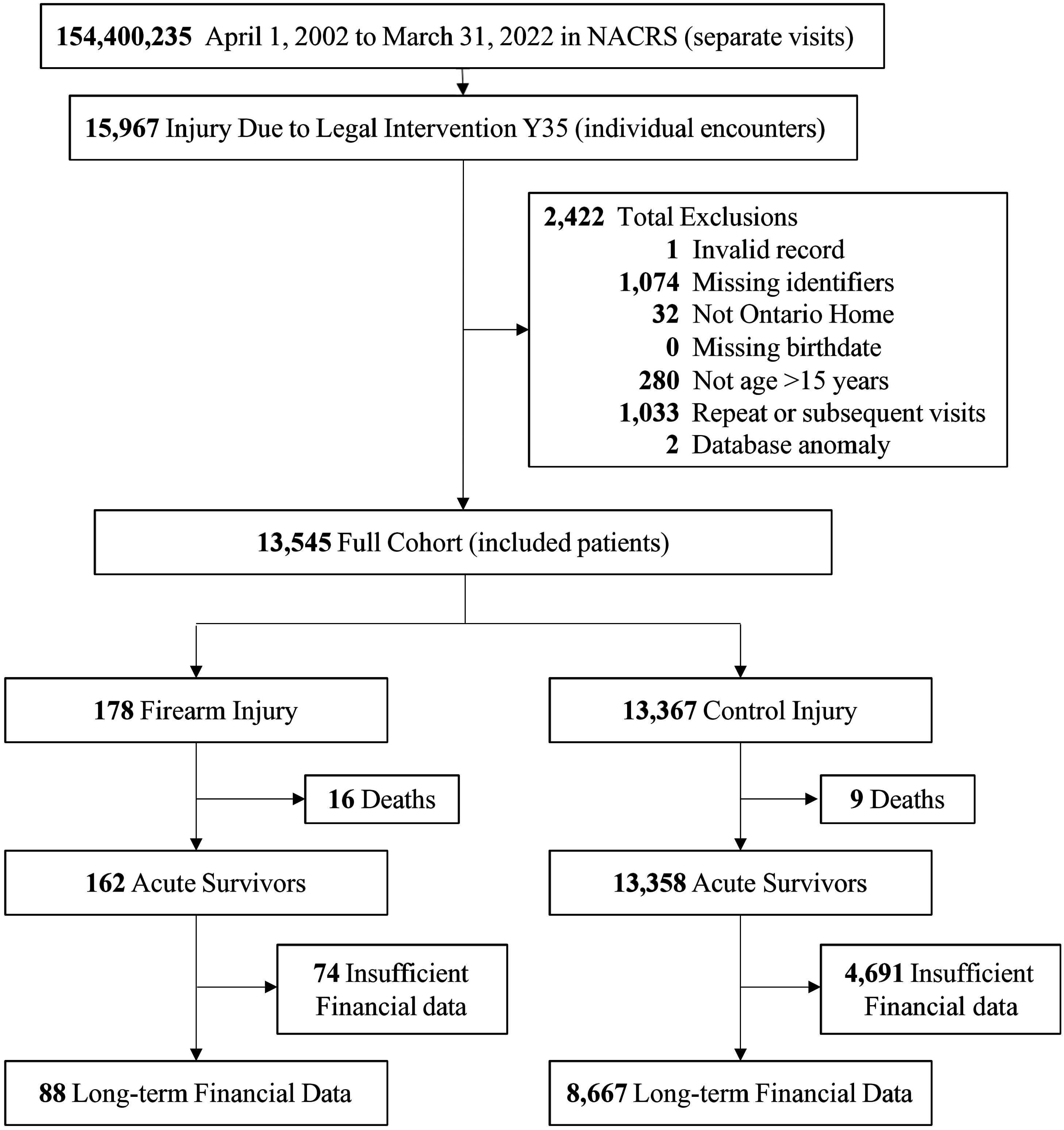

Flow diagram depicting strategy for selecting and classifying study patients. Bold digits show numbers at each point and internal text denotes criteria for including individuals. Counts of exclusions shown as horizontal diversions. Abbreviation NACRS denotes National Ambulatory Care Reporting System for emergency patients. Long-term denotes 5 complete years of follow-up.

© 2023 Raza S et al. *JAMA Network Open.*

eTable 1. Description of Full Cohort

			Firearm *	Control #
			(n = 178)	(n = 13,367)
DEMOGRAPHIC				
Age	≤ 29 years		69 (38.8%)	5,435 (40.7%)
	≥ 30 years		109 (61.2%)	7,932 (59.3%)
Sex	male		166 (93.3%)	11,471 (85.8%)
	female		12 (6.7%)	1,896 (14.2%)
Home	urban		150 (84.3%)	11,952 (89.4%)
	rural		28 (15.7%)	1,415 (10.6%)
Socioeconomic quintile ‡	highest		19 (10.7%)	1,514 (11.3%)
	next to highest		24 (13.5%)	1,921 (14.4%)
	middle		24 (13.5%)	2,326 (17.4%)
	next to lowest		52 (29.2%)	2,819 (21.1%)
	lowest		59 (33.1%)	4,787 (35.8%)
ACUTE INCIDENT				
Night time	yes		69 (38.8%)	5,061 (37.9%)
	no		109 (61.2%)	8,306 (62.1%)
Weekend	yes		50 (28.1%)	4,501 (33.7%)
	no		128 (71.9%)	8,866 (66.3%)
PAST YEAR HEALTHCARE				
Mental health diagnosis †	yes		86 (48.3%)	5,359 (40.1%)
	no		92 (51.7%)	8,008 (59.9%)
Substance misuse diagnosis §	yes		33 (18.5%)	2,633 (19.7%)
	no		145 (81.5%)	10,734 (80.3%)
≥7 outpatient visits	yes		48 (27.0%)	4,281 (32.0%)
	no		130 (73.0%)	9,086 (68.0%)
Emergency visit	yes		96 (53.9%)	7,267 (54.4%)
	no		82 (46.1%)	6,100 (45.6%)

			Firearm *	Control #
			(n = 178)	(n = 13,367)
Hospital admission	yes		21 (11.8%)	1,248 (9.3%)
	no		157 (88.2%)	12,119 (90.7%)
Total healthcare costs ¶	mean ± std dev		$5,384 ±10,094	$5,179 ±15,457
	median (IQR)		$1,105 (271-5,672)	$953 (248-3,870)

© 2023 Raza S et al. *JAMA Network Open.*

eTable 2. Description of Long-Term Cases

			Firearm *	Control #
			(n = 88)	(n = 8,667)
DEMOGRAPHIC				
Age	≤ 29 years		38 (43.2%)	3,784 (43.7%)
	≥ 30 years		50 (56.8%)	4,883 (56.3%)
Sex	male		#	7,538 (87.0%)
	female		#	1,129 (13.0%)
Home	urban		77 (87.5%)	7,802 (90.0%)
	rural		11 (12.5%)	865 (10.0%)
Socioeconomic quintile ‡	highest		12 (13.6%)	969 (11.2%)
	next to highest		12 (13.6%)	1,331 (15.4%)
	middle		11 (12.5%)	1,534 (17.7%)
	next to lowest		28 (31.8%)	1,847 (21.3%)
	lowest		25 (28.4%)	2,986 (34.5%)
ACUTE INCIDENT				
Night time	yes		35 (39.8%)	3,232 (37.3%)
	no		53 (60.2%)	5,435 (62.7%)
Weekend	yes		27 (30.7%)	3,041 (35.1%)
	no		61 (69.3%)	5,626 (64.9%)
PAST YEAR HEALTHCARE				
Mental health diagnosis †	yes		42 (47.7%)	3,091 (35.7%)
	no		46 (52.3%)	5,576 (64.3%)
Substance misuse diagnosis §	yes		14 (15.9%)	1,336 (15.4%)
	no		74 (84.1%)	7,331 (84.6%)
≥7 outpatient visits	yes		19 (21.6%)	2,549 (29.4%)
	no		69 (78.4%)	6,118 (70.6%)
Emergency visit	yes		39 (44.3%)	4,364 (50.4%)
	no		49 (55.7%)	4,303 (49.6%)

© 2023 Raza S et al. *JAMA Network Open.*

			Firearm *	Control #
			(n = 88)	(n = 8,667)
Hospital admission	yes		11 (12.5%)	692 (8.0%)
	no		77 (87.5%)	7,975 (92.0%)
Total healthcare costs ¶	mean ± std dev		4,002 ± 7,779	3,787 ± 12,310
	median (IQR)		1,086 (239-4,000)	735 (196-2,802)

© 2023 Raza S et al. *JAMA Network Open.*
