# Supplementary material for: Costs for Long-Term Health Care After a Police Shooting in Ontario, Canada
Source: JAMA Netw Open. 2023 Sep 28;6(9):e2335831. doi: 10.1001/jamanetworkopen.2023.35831 (PMC10539992; doi:10.1001/jamanetworkopen.2023.35831)
Supplement: Supplement 2. — Data Sharing Statement [file jamanetwopen-e2335831-s002.pdf]

Data Sharing Statement

Raza. Costs for Long-Term Health Care After a Police Shooting in Ontario, Canada. *JAMA Netw Open*. Published September 28, 2023. doi:10.1001/jamanetworkopen.2023.35831

Data
Data available: No

Additional Information
Explanation for why data not available: Patient privacy laws prohibit from making individual-level data publicly available. Aggregate data are shown in the paper and appendix. Researchers interested in replicating or extending the work can seek access to individual-level data through the Institute for Clinical Evaluative Sciences (contact http://www.ices.on.ca).
